# Characteristics of Patients with Obstructive Sleep Apnea at High Risk for Cardiovascular Disease

**DOI:** 10.3390/medicina57111265

**Published:** 2021-11-18

**Authors:** Izolde Bouloukaki, Michail Fanaridis, Georgios Stathakis, Christina Ermidou, Eleftherios Kallergis, Violeta Moniaki, Eleni Mauroudi, Sophia E. Schiza

**Affiliations:** 1 Sleep Disorders Center, Department of Respiratory Medicine, Medical School, University of Crete, 71110 Heraklion, Greece; michfana@gmail.com (M.F.); stathakisgg@gmail.com (G.S.); christina_ermidou@hotmail.com (C.E.); vmoniaki@yahoo.gr (V.M.); elenima23@hotmail.com (E.M.); schiza@med.uoc.gr (S.E.S.); 2 Department of Cardiology, University Hospital of Heraklion, 71110 Heraklion, Greece; ekallergis@med.uoc.gr

**Keywords:** obstructive sleep apnea, cardiovascular disease, symptoms, co-morbidities

## Abstract

*Background and Objectives:* To evaluate the influence of obstructive sleep apnea (OSA)-related symptoms on prevalent cardiovascular disease (CVD) in a large clinical population of patients. *Materials and Methods:* A total of 2127 patients (mean age 55 years, 24% women) underwent diagnostic polysomnography and were evaluated using the Epworth sleepiness scale (ESS), the Athens Insomnia Scale (AIS), and the Beck Depression Inventory (BDI). We investigated the predictive value of OSA-associated symptoms for prevalent cardiovascular disease, after adjustment for relevant confounding factors including age, obesity, and co-morbidities. *Results:* Patients with OSA and CVD were older and had a higher Body Mass Index (BMI); the percentage of obese patients was also higher (83% vs. 70%, *p* < 0001). They also had greater neck, waist, and hip circumferences and a higher waist-to-hip ratio. Excessive daytime sleepiness (ESS ≥ 10) [odds ratio (95% CI) 1.112 (0.708–1.748), *p* = 0.64], insomnia symptoms (AIS ≥ 6) [odds ratio (95% CI) 0.748 (0.473–1.184), *p* = 0.21], frequent awakenings [odds ratio (95% CI) 1.599 (1.019–2.508), *p* = 0.06], and nocturia [odds ratio (95% CI) 1.359 (0.919–2.009), *p* = 0.124] were not associated with CVD after adjustment for the previous confounders. On the other hand, depressive symptoms (BDI ≥ 10) independently predicted prevalent CVD [odds ratio (95% CI) 1.476 (1.154–1.887), *p* = 0.002]. Further analysis in subgroups stratified by age, BMI, and gender demonstrated that depressive symptoms predicted prevalent CVD but only in the subgroup of younger (age group < 60 years), obese (BMI group ≥ 30), and male (OR = 1.959, 95% CI = 1.209–3.175, *p* = 0.006) OSA patients. *Conclusions:* OSA patients with CVD were more likely to complain of less typical OSA symptoms and depressive symptoms compared to patients without CVD in this large clinical patient cohort, supportingthecomplexity and heterogeneityof OSA.

## 1. Introduction

Obstructive sleep apnea (OSA) is a major and under-recognized public health problem. In fact, OSA has a high prevalence, comparable to that of other chronic respiratory diseases, such as asthma and chronic obstructive pulmonary disease [1,2]. Furthermore, its prevalence seems to be increasing in epidemiological studies over time, probably due to different diagnostic tests, definitions of events, study designs, and the effect of the increasing rates of obesity and other co-morbidities, as well as of increased human longevity [3,4]. In addition, untreated OSA is associated with numerous long-term health consequences, has a negative impact on quality of life and cognitive function, and has even been shown to have a causative role in traffic accidents, resulting in injury and fatality [5,6]. The total burden of OSA is indicatedby the large number of subjects affected multiplied by the cost of adverse consequences that can be attributed to OSA [7]. Therefore, OSA appears to represent a vast economic burden of billions of dollars per year, which is analogous to that of other chronic diseases [8].

Observational studies have demonstrated a consistent association of OSA with hypertension, coronary heart disease, cardiac arrhythmia, heart failure, and stroke [6]. However, whether OSA is directly implicated in the causal pathway of these disorders or is linked to them through common co-morbidities is not always easily understood. It seems that distinct clinical OSA phenotypes, based on characteristics used in every-day clinical practice, may differ as regards the association of OSA with cardiovascular disease (CVD) [9,10]. Indeed, patients with OSA are heterogeneous with respect to symptoms, pathophysiologic traits, and polysomnographic expression of OSA. It has been suggested that certain subgroups of OSA patients may benefit from targeted therapeutic strategies in terms of prevention of CVD events [11].

With this background, the question that remains is whether an improved understanding of specific prognostic features of OSA patients could identify those at high risk for CVD, with a view towards outcome prediction and risk stratification. Prior studies attempting to characterize OSA phenotypes have shown substantial differences in co-morbidities and clinical presentation among OSA patients across Europe [10]. However, limited data regarding clinically OSA phenotypes of patients at high risk for CVD referred for suspected OSA exist in Greece. Therefore, the aim of our study was to characterize OSA symptom subtypes and assess their association with prevalent CVD in a large clinical population of patients in Southern Greece.

## 2. Materials and Methods

### 2.1. Population

We conducted a single-center, retrospective study of consecutive patients aged ≥ 18 years, who were admitted to the Sleep Disorders Center, Department of Thoracic Medicine, University of Crete Medical School, during a 7-year period (2014–2019) for evaluation of suspected sleep-disordered breathing. Exclusion criteria included missing or erroneous study variables, missing or incomplete sleep questionnaire data, and/or missing medical history. Ethical approval was provided by the University Hospital Ethics Committee.

### 2.2. Data Collection

All patients underwent a detailed evaluation as part of the routine clinical evaluation that included age, measurement of body mass index (BMI), medical history focused on sleep-related symptoms, associated conditions and co-morbidities, smoking history, and alcohol intake. In addition, we performed overnight attended polysomnography (PSG). Subjective daytime sleepiness was assessed usingthe Epworth sleepiness scale (ESS), insomnia with the Athens Insomnia Scale (AIS), and patient’s depressive symptoms with the Beck Depression Inventory (BDI).

Epworth Sleepiness Scale (ESS). The ESS is currently the most widely used subjective test of daytime sleepiness in clinical practice [12]. A score of less than 10 is considered as normal. The higher the score (from 10 to 24), the greater the reported subjective daytime sleepiness.

Athens Insomnia Scale (AIS). The 8-item Athens Insomnia Scale is a self-assessment psychometric instrument based on the diagnostic criteria of the International Classification of Diseases 10th Revision (ICD-10), which has been developed as a tool to evaluate the severity of insomnia [13,14]. A total score is obtained after summing up all responses and ranges between 0 and 24; a cut-off point of ≥6 represents a minimum criterion for the confirmation of insomnia symptoms. A higher AIS score indicates a higher level of insomnia.

Beck Depression Inventory (BDI). This 21-item questionnaire is a widely used and well-validated self-reported inventory of depressive symptoms [15,16,17]. Total scores range from 0 to 63 and represent the sum of the highest levels endorsed on each item. Scores below 10 are considered normal. Scores from 10 through 18 indicate mild to moderate depression, scores from 19 through 29 indicate moderate to severe depression, and scores from 30 through 63 indicate severe depression.

### 2.3. Sleep Study

All patients underwent a single-night full diagnostic polysomnography (PSG) study (Alice 5, Diagnostics System, Respironics, Murrysville, PA, USA). PSG studies were performed and analyzed according to the American Academy of Sleep Medicine (AASM) standard criteria [18].The apnea–hypopnea index (AHI), the average number of apneas plus hypopneas expressed per hour of sleep, was used for OSA diagnosis and assessment of its severity. OSA was classified as mild if 5 ≤ AHI < 15, as moderate if 15 ≤ AHI < 30, and as severe if AHI ≥ 30 apneas plus hypopneas per hour of sleep.

### 2.4. Statistical Analysis

Results are presented as mean ± standard deviation (SD) for continuous variables if normally distributed and as median (25th–75th percentile) if not. Qualitative variables are presented as absolute numbers (percentage). For comparisons between groups, a two-tailed *t*-test for independent samples (for normally distributed data) or a Mann–Whitney U test (for non-normally distributed data) was utilized for continuous variables, and the chi-square test for categorical variables. We examined in OSA patients the association of OSA-related symptoms with CVD (dependent variable) after adjustment for various potential explanatory variables, including age (>60 years), BMI(≥30 kg/m^2^), AHI, waist/hip ratio, neck circumference, smoking status, type 2 diabetes, dyslipidemia, chronic obstructive pulmonary disease (COPD), asthma and depression (BDI ≥ 10) (independent variables). For the purpose of this analysis, the term CVD used in logistic regression models referred to any of the following conditions: coronary disease and/or atrial fibrillation and/or stroke and/or heart failure. Age was considered continuously and categorically, as age groups of 18–59 and ≥60 years; BMI was also considered continuously and categorically, as BMI groups of <30 and ≥30 kg/m^2^. Results were considered significant when *p* values were <0.05. Data were analyzed using PAWP 17.0 software (SPSS Inc., Chicago, IL, USA).

## 3. Results

### 3.1. Patients

**Figure 1:**
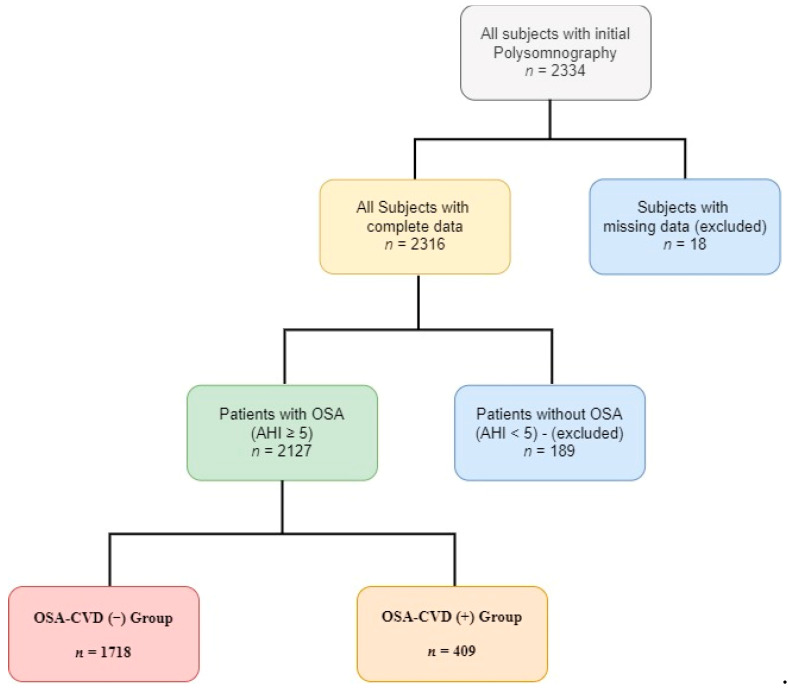
Flow chart of the study.

A total of 2127 patients (mean age 55 years, 26% women) was analyzed (Figure 1). The baseline characteristics of the study population, compared according to CVD prevalence, are shown in Table 1. Overall, 19% of OSA patients had a history of CVD [coronary heart disease (11%) and/or atrial fibrillation (5%) and/or stroke (2%) and/or heart failure (5%)]. Patients with OSA and CVD were older and had a higher BMI; the percentage of obese patients was also higher (83 vs. 70%, *p* < 0.001). They also had higher neck, waist and hip circumference and a higher waist-to-hip ratio. The rate of cigarette (*p* < 0.001) consumption (former plus current smokers) was also higher in OSA patients with CVD.

Co-morbidities prevalence in the whole sample varied between 5% for heart failure and atrial fibrillation and 48% for hypertension. There were differences in the way co-morbidities presented in the two groups. The percentages of patients with hypertension, diabetes mellitus, COPD, and dyslipidemia were higher (*p* < 0.001) in the OSA-CVD group, whereas the rates of asthma, hypothyroidism, and depression were similar in the two groups (*p* > 0.05).

Table 2 and Table 3 show group differences in terms of PSG features and clinical manifestations. A moderate to severe OSA diagnosis was higher in the OSA-CVD group compared to OSA patients without CVD (97 vs. 85%, *p* < 0.001). Indices of OSA severity, such as AHI, AHΙ in REM, oxygen desaturation index (ODI), and total sleep time spent with SaO_2_ < 90% (TST90) were also higher in the OSA-CVD group.

As shown in Table 3, there was no difference in snoring, witnessed apneas, morning headaches, driving problems, and sleepiness and insomnia symptoms between the groups. However, frequent awakenings, nocturia, and depressive symptoms were more commonly observed in the OSA-CVD group.

### 3.2. CVD Association with OSA Symptoms

In Table 4, a multiple stepwise logistic regression analysis of the relationship between CVD and various independent variables is shown. BDI score ≥ 10, indicative of depressive symptoms, was a significant predictor of prevalent CVD after adjustment for confounders (odds ratio (95% CI) 1.505 (1.113–2.036), *p* = 0.008]. However, age > 60 years, male gender, and presence of hypertension and type 2 diabetes were associated with greater odds for CVD compared to depressive symptoms. Moreover, excessive daytime sleepiness (ESS ≥ 10) [odds ratio (95% CI) 1.112 (0.708–1.748), *p* = 0.64) and insomnia symptoms (AIS ≥ 6) [odds ratio (95% CI) 0.748 (0.473–1.184), *p* = 0.21), frequent awakenings (odds ratio (95% CI) 1.599 (1.019–2.508), *p* = 0.06], and nocturia (odds ratio (95% CI) 1.359 (0.919–2.009), *p* = 0.124] were not associated with CVD after adjustment for previous confounders.

### 3.3. Subgroup Analysis by Age, BMI, and OSA Severity

Further analysis in subgroups stratified by age, BMI, and gender demonstrated that depressive symptoms predicted prevalent CVD but only in the subgroup of younger (age group < 60 years), obese (BMI group ≥ 30), and males (OR = 1.959, 95% CI = 1.209–3.175, *p* = 0.006). Additional analysis showed that the OSA severity group was found to influence the predictive value of depressive symptoms on prevalent CVD, as BDI ≥ 10 was a significant predictor only in the moderate to severe OSA group (OR = 1.652, 95% CI = 1.211–2.252, *p* = 0.002).

## 4. Discussion

In our study, which analyzed data froma large database of patients, we tried to extend previous observations and clarify some controversies regarding the symptom profile of patients with OSA and cardiovascular disease. We found that depressive symptoms in OSA patients were among the distinguishing independent predictive factors of CVD, along with older age, male gender, hypertension, and diabetes type 2. We were also able to define a distinct clinical phenotype of younger, obese male, with moderate to severe OSA in which the depressive phenotype predicted mainly CVD. On the other hand, the report of snoring, apneas, frequent awakenings, nocturia, sleepiness, and insomnia symptoms did not show a significant independent association with CVD. The present findings support the complexity and heterogeneity of OSA and the hypothesis that patient with OSA can be categorized into distinct clinically prognostic subgroups associated with increased CVD risk.

Previous studies have shown multiple phenotypes, with different symptom-based subtypes of OSA associated with prevalent CVD [9,10,19,20,21,22]. Three primary generally accepted subtypes have been identified, including patients with minimally or no symptoms, patients with the traditional OSA symptom of excessive daytime sleepiness (EDS), and patients with complaints of insomnia symptoms. These symptoms were also examined in our analysis through the widely used ESS score and the AIS, a questionnaire developed and validated in a Greek population. However, no influence of ESS or AIS score on CVD was noted in our population, questioning if the ESS or AIS tools are sufficient, when employed alone, to characterize the excessive sleepiness or insomnia phenotype of OSA patients at increased risk for cardiovascular events. Furthermore, it also remains unclear if sleepiness or insomnia symptoms are associated with an increase of prevalence in CVD in OSA patients [23,24].

Although no significant effect of these symptoms on CVD prevalence was found, the current study found an additional characteristic of OSA patients at high risk for CVD, defined by depressive symptoms. The presence of depressive symptoms was associated with a 1.5-time risk increase of CVD, and the association significantly persisted in older, males, and obese patients. The relationship between depressive symptoms and increased CVD risk has been studied before in the general population, with a recent large, population-based cohort study reporting that adults with depressive symptoms were associated with an increased risk of incident CVD and mortality in countries at all levels of development [25]. A strong bidirectional relationship has been reported between OSA and depression, with each disease influencing the development of the other [26]; however, to the best of our knowledge, no data exist examining the predictive value of depression or depressive symptoms on CVD risk in OSA patients. Another important finding of our study is that, although a clinical diagnosis of depression was documented in 12% of the patients, a considerable percentage of patients (43%) reported depressive symptoms (BDI ≥ 10), suggesting that health care professionals face challenges in detecting and diagnosing depressive disorders [27]

In our study, the effect of depressive symptoms on prevalent CVD was more prominent in younger and obese male patients with OSA. Therefore, understanding in which age, gender, and BMI group depressive symptoms increase the risk for CVD becomes significant in OSA patients, who may thus have the potential to benefit more than others from a more intensive CVD risk management and Positive airway pressure (PAP) treatment. Nevertheless, future investigations are needed to enhance the categorization of OSA patients, choose a favorable treatment option for each category, and achieve precision-based medicine for these patients [28].

Certain limitations of the present study must be addressed. First, the subjects were enrolled based on a clinical referral to the sleep center, a factor that potentially limits the ability to generalize our findings to other populations. Second, given that our study was cross-sectional and data were evaluated retrospectively, causal inferences are precluded. Furthermore, we did not record sleep habits and duration, which can act as confounding factors for symptoms. Finally, future longitudinal research is suggested to validate the results of this cross-sectional analysis. However, a particular strength of our study is the large population examined in this sleep laboratory study.

## 5. Conclusions

In conclusion, our study intended to address important clinical questions and challenges regarding whether clinical phenotypes of OSA, obtained by means of symptomprofiles, subjectively determined with ESS, AIS, and BDI, may be related to an increased risk for CVD. According to our data regarding a sleep clinic population of Crete, OSA patients reporting insomnia-like symptoms and/or sleepiness do not represent a phenotype at risk for CVD. However, depressive symptoms subjectively measured with BDI were an explanatory variable of the presence of CVD, especially in younger and obese male patients with OSA. A better knowledge of clinical OSA phenotypes will help to improve the awareness and diagnosis of CVD in OSA patients and promote the development and availability of therapeutic options that take into account these phenotypes.

## Figures and Tables

**Table 1:** Characteristics of the study population.

	Total Population According to CVD			
	All OSA Patients (*n* = 2127)	OSA-CVD (−) Group (*n* = 1718)	OSA-CVD (+) Group (*n* = 409)	*p* Value
**Demographics**				
Age(Years)	55 ± 14	54 ± 14	64 ± 12	<0.001
Age ≥ 60 years	851 (40%)	567 (33%)	274 (67%)	<0.001
BMI (kg/m^2^)	33 ± 7	34 ± 7	35 ± 6	0.002
BMI ≥ 30	1531 (72%)	1202 (70%)	339 (83%)	<0.001
Neck circumference (cm)	43 ± 5	42 ± 5	43 ± 4	<0.001
Waist circumference (cm)	115 ± 15	114 ± 16	119± 13	<0.001
Hip circumference (cm)	115 ± 15	115 ± 16	118 ± 14	<0.001
Waist/Hipcircumference ratio	1.0 ± 0.16	1.0 ± 0.18	1.0 ± 0.08	0.57
**Smoking status**				
Never, *n* (%)	681 (32%)	567 (33%)	106 (26%)	
Current Smoking, *n* (%)	617 (29%)	533 (31%)	82 (20%)	
Former, *n* (%)	820 (39%)	618 (36%)	221 (54%)	<0.001
Pack-years	23 (0, 50)	20 (0, 45)	35 (0, 45)	<0.001
**Co-morbidities**				
Hypertension	1021 (48%)	722 (42%)	303 (74%)	<0.001
Diabetes type II	425 (20%)	275 (16%)	139 (34%)	<0.001
COPD	362 (17%)	241 (14%)	127 (31%)	<0.001
Bronchial asthma	149 (7%)	104 (6%)	29 (7%)	0.63
Hypothyroidism	319 (15%)	258 (15%)	61 (15%)	0.80
Dyslipidemia	872 (41%)	653 (38%)	213 (52%)	<0.001
Depression	255 (12%)	206 (12%)	62 (15%)	0.08

**Table 2:** Polysomnography characteristics of the study population.

	Total Population According to CVD			
	All OSA Patients (*n* = 2127)	OSA-CVD (−) Group (*n* = 1718)	OSA-CVD (+) Group (*n* = 409)	*p* Value
TRT (min)	421 ± 108	420 ± 117	424 ± 54	0.45
TST (min)	258 ± 61	261 ± 61	245 ± 61	<0.001
Sleepefficiency, %	62 ± 12	63 ± 12	58 ± 13	<0.001
WASO (min)	110 (91, 142)	107 (88, 136)	125 (88, 136)	<0.001
Sleep Latency	41 (26, 65)	39 (26, 63)	47 (26, 63)	<0.001
REM Latency	255 ± 83	253 ± 82	264 ± 86	0.015
NREM (%)	90 ± 6	90 ± 7	91 ± 3	0.001
SWS (%)	7 (5, 9)	7 (6, 9)	6 (6, 9)	<0.001
REM(%)	9 (7, 11)	9 (7, 12)	8 (7, 12)	<0.001
AHI	36 (21, 63)	34 (20, 62)	43 (20, 62)	<0.001
REM AHI	48 ± 28	47 ± 28	54 ± 26	<0.001
AI	46 ± 14	46 ± 14	49 ± 13	<0.001
ODI	40 (24, 66)	38 (22, 65)	48 (22, 65)	<0.001
Mean SaO_2_	92 (90, 93)	92 (90, 94)	91 (90, 94)	<0.001
Lowest SaO_2_	80 (74, 84)	80 (75, 84)	78 (75, 84)	<0.001
TST90 (min)	59 (19, 131)	51 (16, 122)	86 (16, 122)	<0.001
**Severity of OSA (%)**				
5 ≤ AHI < 15	278 (13%)	265 (15%)	13 (3%)	
15 ≤ AHI < 30	543 (26%)	445(26%)	98 (24%)	
AHI ≥ 30	1306 (61%)	1008 (59%)	298 (73%)	<0.001

**Table 3:** Nocturnal and diurnal symptoms of the study population.

	Total Population According to CVD			
	All OSA Patients (*n* = 2127)	OSA-CVD (−) Group (*n* = 1718)	OSA-CVD (+) Group (*n* = 409)	*p* Value
**Nocturnal Symptoms**				
Snoring	2084 (98%)	1683 (98%)	404 (99%)	0.413
Witnessed apneas	2084 (98%)	1667 (97%)	405 (99%)	0.042
Athens Insomnia Scale Score	8 ± 5	8 ± 5	8 ± 5	0.79
Athens Insomnia Scale Score ≥ 6 (%)	1426 (67%)	1168 (68%)	249 (61%)	0.12
Frequentawakenings	1723 (81%)	1374 (80%)	356 (87%)	0.001
Nocturia	1617 (76%)	1271 (74%)	344 (84%)	<0.001
**Diurnalsymptoms**				
ESS score	11 ± 5	10 ± 5	11 ± 5	0.39
ESS ≥ 10	1170 (55%)	928 (54%)	233 (57%)	0.26
ESS ≥ 16	383 (18%)	309 (18%)	74 (18%)	0.94
Morningheadache	404 (19%)	326 (19%)	82 (20%)	0.45
Driving Problems	447 (21%)	361 (21%)	82 (20%)	0.69
BDI score	8 (4, 14)	8 (4, 13)	10 (4, 13)	<0.001
BDI ≥ 10	915 (43%)	721 (42%)	213 (52%)	0.005

**Table 4:** Multiple stepwise logistic regression analysis of the relationship between CVD and various independent variables.

	B	S.E.	*p*-Value	OR (95%CI)
Males versus Females	0.506	0.207	0.014	1.659 (1.106–2.487)
Age > 60 years	0.905	0.156	<0.001	2.471 (1.819–3.357)
Body mass index ≥ 30	0.367	0.197	0.062	1.443 (0.982–2.122)
Smoking (Current/Former)	0.295	0.181	0.103	1.342 (0.942–1.913)
Hypertension	0.898	0.166	<0.001	2.453 (1.771–3.399)
Type 2 diabetes	0.418	0.176	<0.018	1.519 (1.075–2.146)
Dyslipidaemia	0.215	0.151	0.154	1.240 (0.922–1.668)
Moderate/Severe OSA	0.658	0.349	0.03	1.931 (0.975–3.826)
BDI ≥ 10	0.409	0.154	0.008	1.505 (1.113–2.036)

## References

[1] Benjafield A.V., Ayas N.T., Eastwood P.R., Heinzer R., Ip M.S.M., Morrell M.J., Nunez C.M., Patel S.R., Penzel T., Pépin J.-L. (2019). Estimation of the global prevalence and burden of obstructive sleep apnoea: A literature-based analysis. Lancet Respir. Med..

[2] Xie M., Liu X., Cao X., Guo M., Li X. (2020). Trends in prevalence and incidence of chronic respiratory diseases from 1990 to 2017. Respir. Res..

[3] Jehan S., Myers A.K., Zizi F., Pandi-Perumal S.R., Jean-Louis G., McFarlane S.I. (2018). Obesity, obstructive sleep apnea and type 2 diabetes mellitus: Epidemiology and pathophysiologic insights. Sleep Med. Disord. Int. J..

[4] Umbro I., Fabiani V., Fabiani M., Angelico F., Del Ben M. (2020). Association between non-alcoholic fatty liver disease and obstructive sleep apnea. World J. Gastroenterol..

[5] Morsy N., Farrag N., Zaki N.F., Badawy A.Y., Abdelhafez S.A., El-Gilany A.-H., El Shafey M.M., Pandi-Perumal S.R., Spence D.W., BaHammam A.S. (2019). Obstructive sleep apnea: Personal, societal, public health, and legal implications. Rev. Environ. Health.

[6] Garvey J.F., Pengo M.F., Drakatos P., Kent B.D. (2015). Epidemiological aspects of obstructive sleep apnea. J. Thorac. Dis..

[7] American Academy of Sleep Medicine (2016). Hidden Health Crisis Costing America Billions: Underdiagnosing and Undertreating Obstructive Sleep Apnea Draining Health Care System.

[8] Knauert M., Naik S., Gillespie M.B., Kryger M. (2015). Clinical consequences and economic costs of untreated obstructive sleep apnea syndrome. World J. Otorhinolaryngol. Head Neck Surg..

[9] Saaresranta T., Hedner J., Bonsignore M.R., Riha R.L., McNicholas W.T., Penzel T., Anttalainen U., Kvamme J.A., Pretl M., Sliwinski P. (2016). Clinical phenotypes and comorbidity in European sleep apnoea patients. PLoS ONE.

[10] Anttalainen U., Grote L., Fietze I., Riha R.L., Ryan S., Staats R., Hedner J., Saaresranta T. (2019). Insomnia symptoms combined with nocturnal hypoxia associate with cardiovascular comorbidity in the European sleep apnea cohort (ESADA). Sleep Breath..

[11] Quan W., Zheng D., McEvoy D., Barbe F., Chen R., Liu Z., Loffler K., Lorenzi-Filho G., Luo Y., Mukherjee S. (2018). High risk characteristics for recurrent cardiovascular events among patients with obstructive sleep apnoea in the SAVE study. EClinicalMedicine.

[12] Johns M.W. (1991). A new method for measuring daytime sleepiness: The Epworth sleepiness scale. Sleep.

[13] Soldatos C.R., Dikeos D.G., Paparrigopoulos T.J. (2000). Athens Insomnia Scale: Validation of an instrument based on ICD-10 criteria. J. Psychosom. Res..

[14] Soldatos C.R., Dikeos D.G., Paparrigopoulos T.J. (2003). The diagnostic validity of the Athens Insomnia Scale. J. Psychosom. Res..

[15] Beck A.T., Beamesderfer A. (1974). Assessment of depression: The Depression Inventory. Anxiety Disord..

[16] Beck A.T., Steer R.A., Carbin M.G. (1988). Psychometric properties of the Beck Depression Inventory: Twenty-five years of evaluation. Clin. Psychol. Rev..

[17] Richter P., Werner J., Heerlein A., Kraus A., Sauer H. (1998). On the validity of the Beck Depression Inventory. Psychopathology.

[18] Berry R.B., Brooks R., Gamaldo C.E., Harding S.M., Marcus C., Vaughn B.V. (2016). The AASM manual for the scoring of sleep and associated events: Rules, terminology and technical speci-fications. Am. Acad. Sleep Med..

[19] Mazzotti D.R., Keenan B.T., Lim D.C., Gottlieb D.J., Kim J., Pack A.I. (2019). Symptom subtypes of obstructive sleep apnea predict incidence of cardiovascular outcomes. Am. J. Respir. Crit. Care Med..

[20] Ye L., Pien G.W., Ratcliffe S., Björnsdottir E., Arnardottir E.S., Pack A., Benediktsdottir B., Gislason T. (2014). The different clinical faces of obstructive sleep apnoea: A cluster analysis. Eur. Respir. J..

[21] Keenan B.T., Kim J., Singh B., Bittencourt L., Chen N.-H., Cistulli P.A., Magalang U.J., McArdle N., Mindel J.W., Benediktsdottir B. (2018). Recognizable clinical subtypes of obstructive sleep apnea across international sleep centers: A cluster analysis. Sleep.

[22] Kim J., Keenan B.T., Lim D.C., Lee S.K., Pack A.I., Shin C. (2018). Symptom-based subgroups of Koreans with obstructive sleep apnea. J. Clin. Sleep Med..

[23] Frangopoulos F., Zannetos S., Nicolaou I., Economou N.T., Adamide T., Georgiou A., Nikolaidis P.T., Rosemann T., Knechtle B., Trakada G. (2021). The complex interaction between the major sleep symptoms, the severity of obstructive sleep apnea, and sleep quality. Front. Psychiatry.

[24] Ogilvie R.P., Lakshminarayan K., Iber C., Patel S., Lutsey P.L. (2018). Joint effects of OSA and self-reported sleepiness on incident CHD and stroke. Sleep Med..

[25] Rajan S., McKee M., Rangarajan S., Bangdiwala S., Rosengren A., Gupta R., Kutty V.R., Wielgosz A., Lear S., AlHabib K.F. (2020). Association of symptoms of depression with cardiovascular disease and mortality in low-, middle-, and high-income countries. JAMA Psychiatry.

[26] Pan M.-L., Tsao H.-M., Hsu C.-C., Wu K.-M., Hsu T.-S., Wu Y.-T., Hu G.-C. (2016). Bidirectional association between obstructive sleep apnea and depression. Medicine.

[27] Ferenchick E.K., Ramanuj P., Pincus H.A. (2019). Depression in primary care: Part 1—Screening and diagnosis. BMJ.

[28] Edwards B.A., Redline S., Sands S.A., Owens R.L. (2019). More than the sum of the respiratory events: Personalized medicine approaches for obstructive sleep apnea. Am. J. Respir. Crit. Care Med..

